# Economic burden of symptomatic iron deficiency – a survey among Swiss women

**DOI:** 10.1186/s12905-019-0733-2

**Published:** 2019-02-26

**Authors:** Patricia R. Blank, Yuki Tomonaga, Thomas D. Szucs, Matthias Schwenkglenks

**Affiliations:** 1 University of Zurich, Epidemiology, Biostatistics and Prevention Institute, Hirschengraben 84, 8001 Zurich, Switzerland; 2 University of Basel, Institute of Pharmaceutical Medicine, Klingelbergstrasse 61, 4056 Basel, Switzerland

**Keywords:** Economic impact, Iron deficiency, Misdiagnosis; societal burden

## Abstract

**Background:**

Symptomatic iron deficiency (ID) is a disorder affecting 10–20% of menstruating women. ID is diagnosed by measuring serum ferritin, a protein helping to store iron in the body. A deeper understanding of the association between ID and its societal and economic burden is relevant for patients, physicians, health care decision makers.

**Methods:**

An online household survey was carried out among Swiss women aged 18–50 years suffering from debilitating symptoms due to ID. The data was population-weighted for age and region. The costs of misdiagnosis and the ID-related economic burden (i.e. days of sick leave) from productivity losses on the labor market were determined and extrapolated to the Swiss population. Furthermore, the patient burden was assessed based on quality of life daily measurements.

**Results:**

The total sample included 1010 women who received an ID diagnosis with a blood test in the last 2 years (mean age: 33.5 years). Most named symptoms were “being tired or exhausted” (96.4%) and reduced physical energy level (41.0%). In total, 354 (35.0% of the total sample) patients received an initial diagnosis other than ID. Of those, 46.8% were treated prior to the ID diagnosis with a pharmacological medical therapy or psychotherapy. Extrapolating these numbers to the Swiss female population aged 18–50 years, the direct medical costs would be CHF 78 million (assuming an annual ID incidence of ID diagnosis of 9.5%). On average, 28.5% of participants in the work-force had to take sick leave due to ID symptoms within a period of 2 years (mean: 5.2 days, i.e. 2.6 days/year). The estimated annual indirect costs in Switzerland would be CHF 33 million (human capital approach) or CHF 26 million (friction cost method), respectively. Being exhausted and impaired concentration appear to be the most important factors negatively impacting daily living and hence quality of life.

**Conclusion:**

The societal and economic burden among women due to debilitating symptoms of ID in Switzerland is substantial. Timely, correct diagnosis and treatment of ID may contribute to reducing this burden. Further studies are needed in this area to validate our results.

## Background

Nearly one-third of the non-pregnant women worldwide are affected by anemia, of which iron deficiency (ID) is the primary cause [1, 2]. Symptomatic ID is a nutrient-related disorder which is significantly prevalent in both developing and industrialized countries, affecting about 10–20% of menstruating women [3–5]. In addition to menstrual blood loss, common causes of ID in adults include inadequate dietary intake, chronic blood loss, times of increased need (i.e. pregnancy), vigorous exercise and other underlining diseases [6, 7]. Also, an inability to absorb iron may be play a role [8]. ID can be diagnosed with measuring the serum ferritin, which is a protein that helps to store iron in the body [9]. Treatment for ID includes adding iron-rich foods to the diet and taking iron therapies [5].

Iron deficiency has an impact on the physical performance, work productivity and cognitive function of affected individuals [10]. Beside this, intravenous iron therapy (IV iron) is controversially discussed in Switzerland in clinical journals, among health insurers and in the press. In 2014, the Swiss Medical Board (SMB) has conducted a Health Technology Assessment (HTA) and came to the conclusion that the administration of IV iron seems to be reasonable, especially in severe ID patients [11].

A deeper understanding of the association between ID, quality of life and its societal and economic burden is important for patients, physicians and health care decision makers.

The main objective of the present study was to assess the economic and societal burden of non-pregnant, female ID patients aged 18–50 years in Switzerland. Direct costs for a delayed or incorrect diagnosis or a sub-optimal therapy (i.e. patients diagnosed with other diagnoses before the diagnosis of ID was confirmed) were determined. Furthermore, the indirect costs were quantified for sick leaves of working women due to debilitating symptoms of ID. Patient burden was quantified in terms of presence of symptoms, quality of life and energy level before any ID therapy has started.

## Methods

### Study design

A population based cross-sectional survey was carried out among Swiss females aged 18–50 years (Table 1). The survey was administered during a quantitative 15-min online survey conducted as part of consumer panels in April 2016. Pre-menopausal women aged 18–50 years suffering from ID were included according to specific inclusion criteria. The targeted sample size was 1000 patients.

**Table 1:** Patient characteristics (weighted, rounding errors may occur)

Variable	Total
Net size (N)	1010
Age (Mean in years; 95% CI); Range (in years)	33.5 (32.9; 34.1); 18–50
18–28 years (N, %)	322 (31.9)
29–39 years (N, %)	305 (30.2)
40–50 years (N, %)	383 (37.9)
Region (N; %)	
German speaking	767; 75.9%
French-speaking	243; 24.1%
Civil status (N; %)	
Unmarried	507; 50.2%
Married	403; 39.9%
Widowed	10; 1.0%
Divorced	78; 7.7%
Work time (N; %)	
Working min. 80%	557 (55.2%)
Part-time (less than 80%)	417 (41.3%)
Not working (N; %)	36 (3.5%)
• Studying	• 16; 1.6%
• Housewife	• 14; 1.4%
• Unemployed	• 3; 0.3%
• Other	• 3; 0.3%
Swiss Nationality (N; %)	934 (92.4)
Size of household (N; %)	
Mean, median (persons)	3.21, 3.0
1 Person	118; 11.7%
2 Persons	174; 17.2%
3 Persons	241; 23.8%
4 Persons	330; 32.8%
5 Persons and more	146; 14.4%
Income level per month (N; %)	
≤ CHF 4000	88; 8.7%
CHF 4001 – 6000	165; 16.4%
CHF 6001 – 8000	171; 16.9%
CHF 8001 – 10,000	174; 17.2%
CHF 10,001 – 15,000	106; 10.4%
> 15,000	58; 5.7%
Not reported	246; 24.4%

Within the study sample, there was a slight overrepresentation of 30–39 year old women (28.4%) and an underrepresentation of 18–29 year olds (39.7%; German-speaking and French-speaking) and 40–50 years olds (31.9%; French-speaking), respectively. In Switzerland,

The sample was population-weighted according to age and region to represent the Swiss female population between 18 to 50 years.

The questionnaire included screening questions to identify women who were diagnosed with ID within the last 2 years. Eligible women were provided with a questionnaire on ID symptoms, number of consultations for ID symptoms, the specialization of the physician who diagnosed ID, and how diagnosis was made. It was expected that patients might have been diagnosed with other diseases before they received their ID diagnosis. Considering typical symptoms of ID, patients were asked if they had been diagnosed with depression, burnout, anxiety state, chronic fatigue or others conditions, and whether this lead to treatment with a medical therapy or psychotherapy. Patient burden was assessed with questions regarding the impact on daily living and the subjective energy-level. Related to the burden, the number of unproductive days at work and the number of missed working-days were recorded for women in the work-force. Demographic information was collected upfront as part of the consumer panel.

The consumer panel consists of more than 130,000 active, recruited panelists between 15 to 74 year-old from all three language areas in Switzerland. The panelists are actively recruited as part of representative, telephone studies with landline telephone numbers and randomly generated mobile telephone numbers. The participation in the panel is voluntary, non-binding and anonymously. At the beginning of the survey, participants, consent to take part is explicitly asked. No specific ethical approval was required for this type of research, as no identifiers were collected. The survey among consumer panels was conducted in line with the Swiss law of data protection and the national and international codes on market and social research guidelines which also include ethical principles [12–16].

### Statistics

Baseline characteristics were summarized as numbers and percentages, means, medians for categorical, normally distributed and non-normally distributed continuous data, respectively.

Discrete numeric/ continuous variables were analyzed with a t-test (for non-skewed data) or a nonparametric test (e.g. Mann-Whitney U test for skewed data). Bivariate associations of categorical variables were assessed with the chi-squared test.

Associations between energy level and present misdiagnosis or sick leave were assessed by Spearman’s correlation coefficient (rho). Correlation coefficients can range from − 1 to + 1 for a perfect negative relationship and a perfect positive relationship, respectively. A value of 0 indicates no correlation. Spearman correlation coefficients were regarded as suitable to indicate probable directions of effects but not to provide reliable estimates of effect size. Therefore, the strength of the correlations shown should be interpreted with caution.

For all statistical tests, two-sided *p*-value < 0.05 was used as the level of statistical significance. Ninety five percent confidence intervals (95% CIs) are reported as appropriate.

The data analysis was carried out with SPSS version 22.

### Costs of misdiagnoses

To assess the impact of misdiagnosis, the direct costs of four conditions (depression, burnout, anxiety state, chronic fatigue) with a possibility for ID-like symptoms were calculated based on per-patient lump sums for each disease (Table 2) [17, 18]. The estimates of per patient costs of mild depression [19], anxiety state [20] stemmed from a recent study analyzing the costs of non-communicable diseases in Switzerland (2011) [17]. The per-patient costs for burnout/ stress in Switzerland was based on an analysis by the Secretariat for Economic Affairs (SECO) for 2003 [21]. The costs for anxiety state stemmed from a recently published study from the UK, given that no data from Switzerland was available [18].

**Table 2:** Patients with a misdiagnosis and related therapies

Misdiagnosis	Frequency	Therapy	Fraction with misdiagnosis and therapy among total sample	Duration of therapy (medical/psychotherapy, in weeks)		
	N; %	N; %	%	Mean	95% CI	Min; Max.
Depression	113; 11.2	71; 62.2	7.0	104	67; 140	0; 780
Burnout	53; 5.3	22; 41.0	2.2	27	5; 50	0; 416
Anxiety state	52; 5.2	30; 56.5	2.9	51	14; 88	0; 780
Chronic fatigue	71; 7.1	20; 27.5	1.9	54	10; 117	0; 624
Others	65; 6.5	31; 47.0	3.0	34	1; 71	0; 1560
Total	354; 35.3	174; 46.8	17.0	–	–	–

The costs from the Swiss publications were corrected for the increase of health care costs over time by the Swiss health insurance index by the Swiss Federal Statistical Office (for the year 2015), given the third-party payer perspective of the analysis [22]. The costs from the UK study for anxiety was corrected for the volume and price (for 2010). The volume correction was based on the nominal and purchasing power per capita health care cost expenditures in UK compared to Switzerland [23]. The price correction compares the prices for a specific intervention in UK and in Switzerland for a specific year [23]. The costs were applied to Switzerland by using a correcting factor for the increase of health care costs based (Swiss health insurance index) [22, 24]. This approach was already used in another study in Switzerland [17, 25].

The per-patient costs were only applied to the fractions of misdiagnosed patients with a prescribed medical or psycho-therapy, as reported in the study sample.

### Economic burden of ID (based on lost working days; indirect costs)

ID-related days of sick leave in the female population in the workforce were assessed and the resulting societal burden of the disease was calculated as indirect costs. The indirect costs of lost working days can e.g. be measured as productivity losses on the labor market, via the human capital approach [26, 27]. This approach assumes that patients who are absent from work due to ID-like symptoms and the respective persons are not available for paid work and not replaced. Under this approach, indirect costs are calculated as the number of lost working-days due to ID multiplied with the mean salary for 1 working day. As an alternative to the human capital approach, the friction cost method has been proposed. This method assumes that contributions of new or already employed workers make partially up for the production losses of the person on sick leave. Therefore, an 80% elasticity for annual labor time versus labor productivity is introduced (the results based on the human capital is multiplied with 0.8) [28, 29].

In Switzerland, a yearly average income of CHF 67,400 (full-time employee) was assumed [30]. Therefore, for the human-capital approach, an average income of CHF 281 per day was anticipated (by assuming a 8.4 h working day, full-time position) [30]. The assumed daily rate corresponded to the opportunity costs of a lost working day. In order to quantify the indirect costs (by the human capital approach), the number of lost working days were multiplied with the daily rate of CHF 281.

## Results

### Sample characteristics

**Fig. 1:**
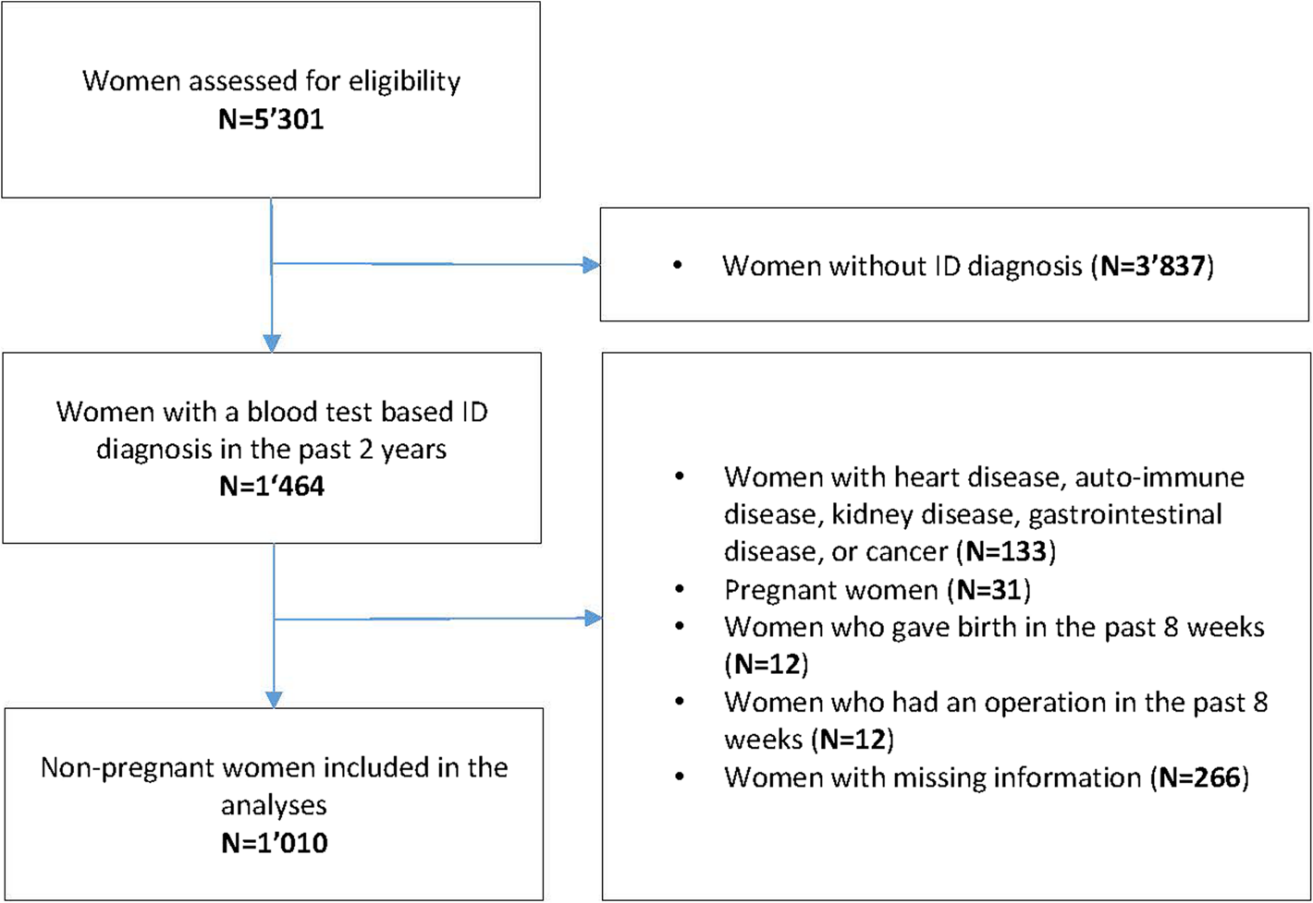
Flowchart of patient enrollment

As illustrated in Fig. 1, out of 5301 invited women, 1010 were eligible and included in the final analyses. The total of 1010 individuals had an ID diagnosis with a diagnostic test within the last 2 years, they had specific symptoms of ID and they were not pregnant, not suffering of a chronic disease or had no operation in the last 8 weeks. Patient characteristics are shown in Table 1. The mean age of the included women was 33.5 years, and only 3.5% reported that they were not working.

In 96.4% of cases, “being tired or exhausted” led to the decision to consult a medical doctor, followed by reduced physical energy level (41.0%) and headache (27.7%). In the mean, it took 28.3 weeks until a medical doctor was consulted due to the named symptoms (median: 12 weeks, range: 0 weeks n – 1040 weeks). In total, 700 (69.3%) of all women mentioned a history of repeated ID diagnosis (mean number of diagnosis: 3.2; median: 2.0).

### ID diagnosis and costs of misdiagnoses due to similar symptoms

#### ID diagnosis

In the mean, patients had to visit a medical doctor for 1.7 times (within a time span of 8.5 weeks) until ID was diagnosed. Most of the ID diagnoses (77.8%) were confirmed by general practitioners (GPs) followed by gynecologists (17.9%) and the hospital (0.9%).

#### Misdiagnosis (initial diagnosis)

A misdiagnosis was assumed in case of an initial diagnosis other than ID, in which case the prescribed treatment other than iron therapy showed a lack of efficacy and in which iron therapy improved symptoms. Table 2 shows the frequency of other diagnoses such as depression, burnout, anxiety state, chronic fatigue and others. In total, 354 (35.0% of the total sample) patients received an initial diagnosis other than ID. Of those, 46.8% were treated prior to the ID diagnosis with a medical therapy or psychotherapy (16.9% of the total sample). The mean duration of the therapy ranged between 34 and 104 weeks.

Comparing patients with a misdiagnosis and without a misdiagnosis shows a statistically significant difference regarding the mean number of consultations (3.0 versus 1.3, *p*-value: < 0.001) and the mean duration of sick leave (9.6 days versus 2.0 days, p-value: < 0.001). This fact would also have an impact on the health care costs but was not incorporated in our analysis.

#### Costs of misdiagnoses

Based on the numbers of patients with an initial diagnosis other than ID and the fraction of patients receiving treatment for the respective disease, the total cost for all misdiagnosed patients within our sample population was determined. The per-patient costs for depression (CHF 5085), burnout (CHF 1424), anxiety state (CHF 1318) or chronic fatigue (CHF 946) were multiplied with the number of misdiagnosed patients within our sample [17, 18]. The total costs of all treated patients ranged between CHF 18,471 for chronic fatigue and CHF 357,385 for depression (Table 2). This would yield in CHF 110 per patient with ID within our sample population. The costs for depression were the major driver (80.2%) of the health care costs in our sample.

**Fig. 2:**
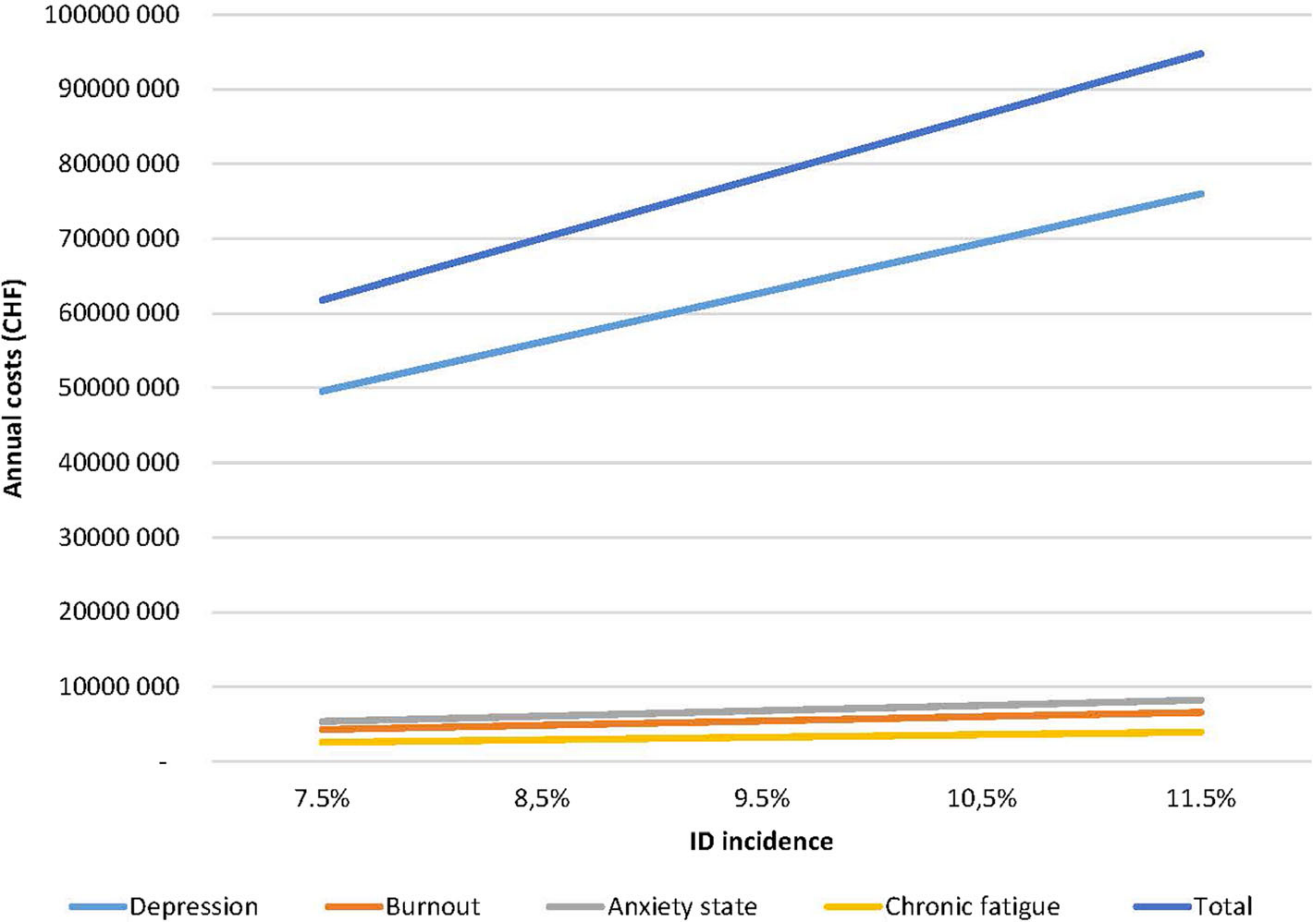
Extrapolation to the Swiss population depending on ID incidence: Total direct costs (CHF)  for misdiagnosis of ID

In order to estimates costs to the Swiss health care system (budget impact), the number of potential patients was identified based on the size of the Swiss female population aged 18–50 years old (*n* = 1,867,768 in 2014) [30]. Based on our sample (1010 women diagnosed with ID during 2 years, of 5301 screened for eligibility), we assumed an annual incidence of ID diagnosis of 9.5% (range for sensitivity analysis, +/− 20%: 7.5–11.5%). This leads to a population-level estimate of *N* = 177,438 diagnosed cases per year (range: 140,083-214,793) [3–5]. According to our observations, a fraction of 14% would receive a misdiagnosis and an underlying therapy for depression, burnout, anxiety state and chronic fatigue (“others” were not taken into account).It is thus expected that 19,545 to 29,969 Swiss female patients per year may be potentially misdiagnosed (Table 3). The direct medical costs resulting therapies would be CHF 78 million (range: CHF 62 million and CHF 95 million; Fig. 2).

**Table 3:** Number (N) of potential misdiagnoses in the entire Swiss female population (aged 18–50 years) depending on iron deficiency incidence

	Incidence of iron deficiency				
	7.5%	8.5%	9.5%	10.5%	11.5%
Depression	9748	11,048	12,348	13,648	14,947
Burnout	3014	3416	3818	4219	4621
Anxiety state	4075	4618	5162	5705	6248
Chronic fatigue	2708	3069	3430	3791	4152
Total	19,545	22,151	24,757	27,363	29,969

### Economic burden of ID due to symptoms (measured as absence from work)

#### Absence from work due to symptoms

Within the sample, 291 (28.8%) women mentioned that they were not able to comply with their daily business (e.g. work, studies, household) due to the named symptoms (mean: 11.8 days; median: 5 days). Within those participants in the workforce, who mentioned that they were not able to comply with their daily business (*N* = 287, 28.7%), they had in the mean 5.2 missed work days over 2 years (hence 2.6 days within 1 year) (Table 4). Within this sub-sample, there were also women with zero missed work days. If only women with ≥1 missed work days due to ID symptoms were taken into account, the mean missed work days were 10.8 (*n* = 138, 14.2% of the total sample). Among housewives, external help was required for 1.2 days (mean).

**Table 4:** Missed work-days among full, part time and non-working participants

	Total sample	Persons not able to comply with work	Persons not able to comply with work	Number of days not able to comply with work	Missed work days^a^	Missed work days^b^	Required external help
	N	N	%	Mean days	Mean days	Mean days	Mean days
Full time (≥ 80%)	557	158	28.4	11.52	3.66	6.75	
Part time (< 80%)	417	119	28.5	12.39	7.33	18.9	
Not working	35	13	37.1	9.18	–	–	1.2
Total	1010	291	28.8	11.8	5.2	10.8	1.2

^a^ including those with 0 missed work days

^b^ excluding those with 0 missed work days

#### Economic burden of ID (measured as absence from work)

In our sample, 974 participants were either full-or part-time workers (96.5%). Currently, the fraction of Swiss female in the workforce is 88.9% among 25 to 54 years old [31]. Hence, in our sample, the proportion of Swiss women working was slightly higher.

Extrapolating these numbers to the Swiss female population between 18 and 50 years (n = 1,867,768), we would assume that 177,438 (range: 140,083 to 214,793) are potentially suffering from ID each year (based on an incidence of ID diagnosis of 9.5%, range: 7.5 to 11.5%). If we only consider women in the work-force, the number would be 157,742 (range: 124,533 to 190,951). In total, we would assume that 45,430 (range: 35,866 to 54,994 depending true incidence) patients would take on an average 2.6 days of sick leave due to debilitating symptoms of ID per year, yielding a total of 118,117 (range: 92,251 to 142,984)days per year.

**Fig. 3:**
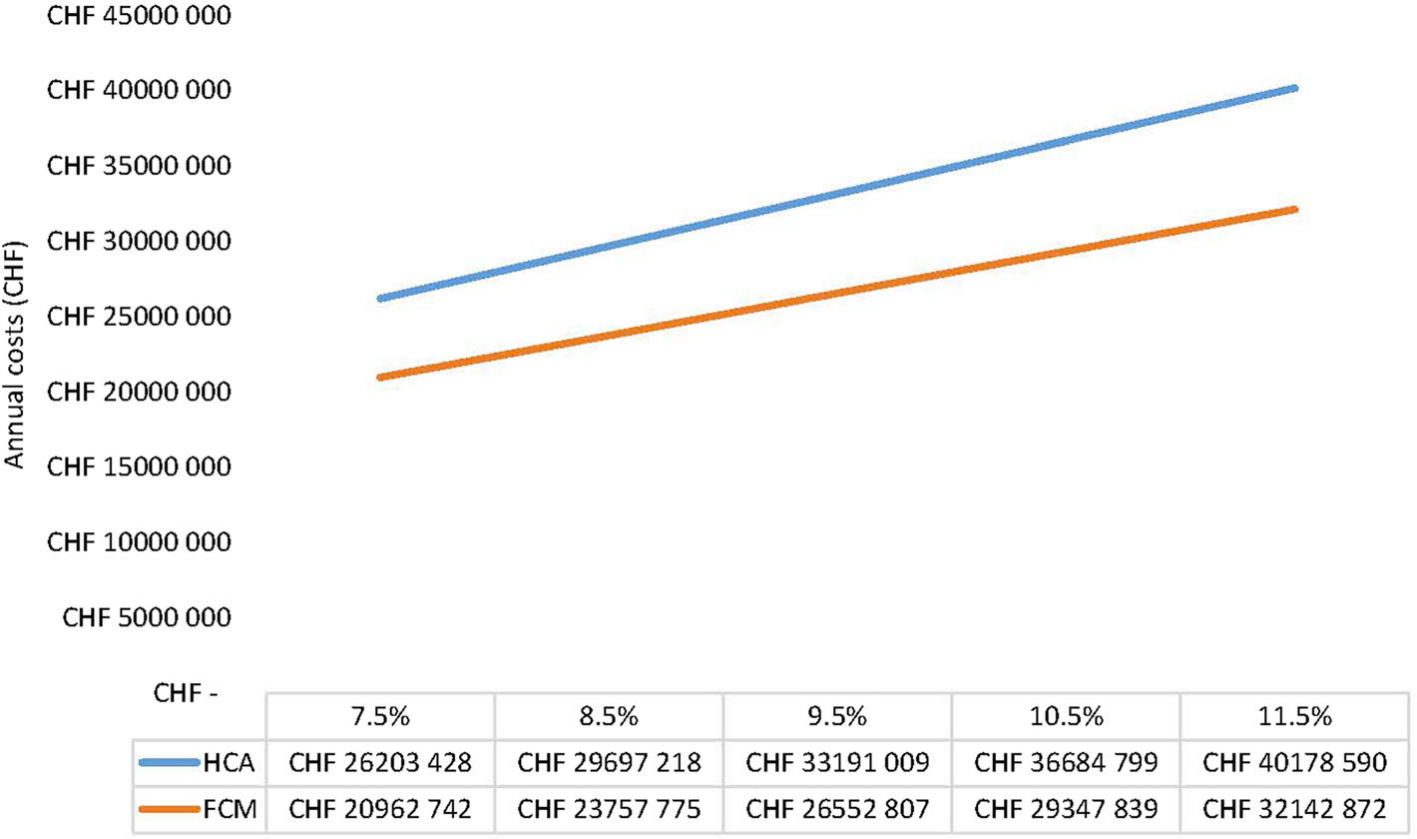
Estimated societal economic burden/ indirect costs of ID patients in Switzerland due to sick leave (HCA: human capital approach; FCM: friction cost method)

Based on an incidence rate of 9.5% (range: 7.5 to 9.5%), the estimated societal economic burden would be CHF 33 million (range: CHF 26 million to CHF 40 million), if calculated with the human capital approach and CHF 26 million (range: CHF 21 million to CHF 32 million) with the friction cost method, respectively (Fig. 3).

### Impact on quality of life

**Fig. 4:**
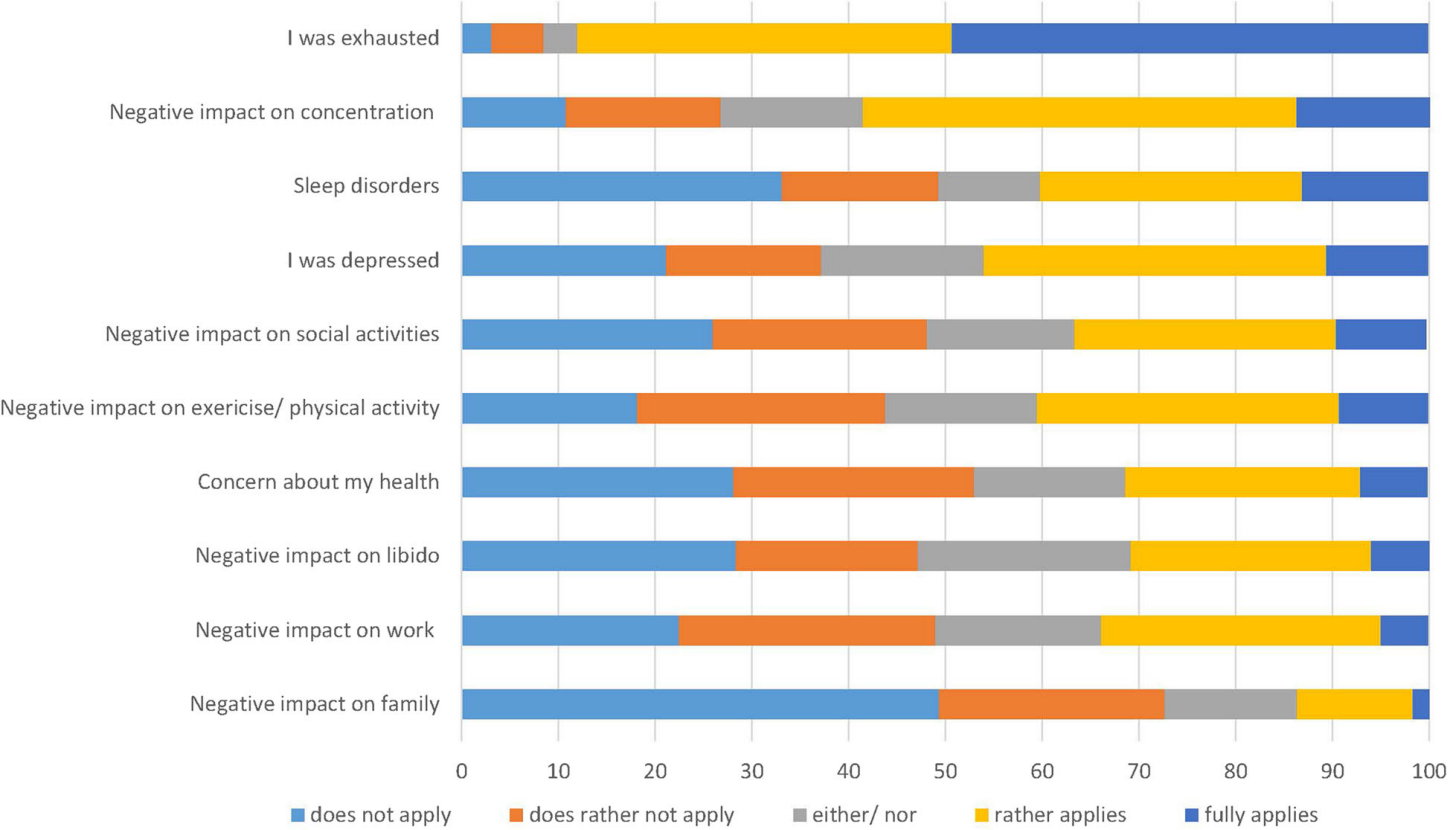
Factors with an impact on quality of life before ID treatment (frequency, %)

To determine the societal burden, patients were asked to rank various aspects of their life into five categories and their energy level (0–100%) before any ID therapy. Being exhausted and impaired concentration appear to be the most important factors impacting daily living negatively and hence quality of life (Fig. 4).

Associations between energy level (before ID diagnosis) and present misdiagnosis or sick leave were assessed by Spearman’s correlation coefficient. A positive, weak correlation between patients without misdiagnoses and mean energy level was shown (rho: 0.14; *p*-value: < 0.001). Furthermore, the analysis indicates a further, but weak correlation between duration of sick leave and lower energy level (rho: − 0.19; *p*-value: 0.01).

## Discussion

Despite its high frequency in non-pregnant women, symptomatic ID is often misdiagnosed as depression, burnout, anxiety or chronic fatigue. This often leads to potentially unnecessary and ineffective treatments, with consequent delay in disease management, postponed patient recovery, and increased healthcare expenditures. Among the 1010 ID patients enrolled in this study, approximatively one third received an initial diagnosis other than ID. Assuming a 9.5% annual incidence of ID diagnosis in Switzerland, we estimate that 24,757 (range ± 20%: 19,545 to 29,969) female patients may initially be misdiagnosed, causing direct medical costs of CHF 78 million (range: CHF 62 million to CHF 95 million). This excludes cases which are misdiagnosed but do not get a correct diagnosis, and thus could not be considered in our survey. Concerning the overall burden of ID in terms of absence of work, we estimate that 45,430 working women in Switzerland take on average 2.6 days of sick leave due to debilitating symptoms of ID per year, yielding in 118,117 days per year. The resulting indirect costs would consequently be CHF 33 million or CHF 27 million using a human capital approach or a friction cost method. This estimate only covers women who received an ID diagnosis within two years, the target population of our survey. This implies an underestimation if one assumes that absences from work are actually independent of ID diagnosis and treatment, i.e. that undiagnosed patients also miss work days due to ID related symptoms.

The analysis of the quality of life showed women suffering from ID often feel exhausted or have concentration problems. Moreover, females receiving a correct, timely ID diagnosis reported higher mean energy levels, suggesting that misdiagnoses have a negative impact on their quality of life.

The results of this study are in line with several works published in the last decades and addressing the problems related to nutrition and to ID. Already in 1987, Arthur et al. suggested that ID is often misunderstood, misdiagnosed and mistreated [32]. Kostopoulou and colleagues published a systematic search of the MEDLINE and EMBASE databases regarding the diagnostic difficulty and error of iron deficiency in primary care practice [33]. They suggested that ID is often considered a simple result of menstruation rather than a potential disease causing entity. In a cost-benefit analysis the effects of nutritional programs on ID and ID anemia was investigated. It has been suggested that medical iron therapy and fortification of food with iron may have a great economic impact by increasing the individual productivity [34].

The present work has several limitations. Firstly, the analyzed sample was enrolled through an online survey. Although nowadays most of the pre-menopausal women aged 18–50 years may easily have access to an online platform, we cannot exclude a selection bias. Secondly, as in all consumer panels, there is a certain risk of information bias. Despite this, consumer surveys are a very effective and fast way of generating data regarding a specific research question. A third limitation is the inclusion of German-speaking and French-speaking women only. Women speaking Italian and other non-national languages were excluded mainly due to the fact that the sample size would have been too small to detect differences with sufficient precision. This may reduce the representativeness of the study results for the entire country, including foreign persons. A fourth limitation is the assumption that the annual incidence of ID diagnosis in non-pregnant Swiss women aged 18–50 years is 9.5% (range: 7.5 to 11.5%), based on a comparison of assessed and eligible women in our survey. Published incidence data are lacking. The assumed range of +/− 20% was chosen to keep the estimate conservative. Recent estimations of the WHO suggested that the prevalence of women aged 15–49 years with anemia in Switzerland may range between 9 and 44% [1]. However, prevalence estimates do not provide a suitable basis for estimating costs of misdiagnosis and indirect costs (societal burden), in combination with our survey results.

An additional limitation is related to the calculation of the costs of misdiagnoses at the national level. It could be assumed that all patients with ID consulting a medical doctor would be treated. This might lead to an overestimation of the costs, since not all patients would wish or receive a medical treatment. Therefore, we applied in our analysis only costs to those patients receiving a therapy in reality, i.e. around 50% of the misdiagnosed patients. This should have reduced the overestimation, at least in part.

In addition, we were not able to rule out any confounding of co-diagnosis of patients primarily diagnosed with a disease other than ID. Some patients might suffer from several diagnoses, e.g. patients diagnosed with depression might also suffer from ID and vice versa. Within the survey, a misdiagnosis was clearly defined as an initial diagnosis with unsuccessful therapies and where ID therapy was successful, but misunderstanding cannot be ruled out.

## Conclusion

The societal and economic burden of sick leave of working women due to debilitating symptoms of ID in Switzerland is substantial. Timely, correct diagnosis and treatment of ID may contribute to reducing this burden. Further studies are needed in this area to validate our results.

## Abbreviations

HTA: Health Technology Assessment
ID: Iron deficiency
SECO: Secretariat for Economic Affairs
WHO: World Health Organization
